# A simple method for electron energy constancy measurement

**DOI:** 10.1120/jacmp.v2i1.2628

**Published:** 2001-01-01

**Authors:** R. Paul King, R. Scott Anderson

**Affiliations:** ^1^ Jeff Anderson Regional Medical Center 1724 23rd Avenue, Bldg. C Meridian Mississippi 39301

**Keywords:** electron, quality assurance

## Abstract

A device is described for use in confirming the energy constancy of clinical electron beams. A wedge shaped absorber is placed over an ionization chamber leading to an energy dependent response. A measurement under the energy filter is divided by a measurement in air to correct for the inherent energy dependence of the chamber. A nearly linear response is demonstrated.

PACS number(s): 87.52.–g, 87.53.–j, 87.66.–a

## I. BACKGROUND

The report of AAPM TG40 (Ref. 1) includes a recommendation that each teletherapy electron beam be evaluated monthly to assure that its penetrative ability remains consistent with commissioning data. The standard is that a percent depth dose (PDD) value near the end of the electron range not shift by more than 2 mm from the value established at commissioning. In principle, it is necessary to measure an entire PDD curve to determine the distance by which an individual PDD value has shifted. In practice, the time required for complete PDD measurement is not conducive to frequent checks. A simple constancy measurement technique is desired.

A clinical electron beam's penetrative ability is described by ${\bar{E}}_{0}$, its mean incident energy. ${\bar{E}}_{0}$ is defined^2^ as 2.33 times the depth in water at which the PDD is equal to 50%. For the purpose of quality assurance, it suffices to verify that ${\bar{E}}_{0}$ is constant.

A common technique is to compare the ionization measured at two depths in the phantom. The first depth is selected to be near $d_{\max}$ and the second depth is selected to be near the reference $d_{50}$. The ratio of these measurements is compared to a reference ratio. There are three drawbacks to this technique: (1) The measurement apparatus must be customized for each individual treatment unit, i.e., the needed arrangement of phantom pieces can be different for two beams, each having the same nominal energy if their $d_{\max}$ or $d_{50}$ values differ; (2) the process is arduous in that the apparatus must be reconfigured prior to each exposure; (3) the measurement result does not lend itself to direct interpretation, i.e., a discrepancy in ionization ratio does not point directly to a corresponding error in beam energy.

Moyer^3^ observed that a wedge‐shaped aluminum block could be used to simultaneously subject an electron beam to different amounts of attenuation. Moyer's technique is to image a wedge‐attenuated beam with a perpendicular film. When the density profile is scanned along the wedged direction, a simulated practical range can be found using graphical analysis. The simulated practical range is cross‐calibrated at commissioning to establish its relationship to the practical range in water. A discrepancy in the simulated practical range points directly to a corresponding energy error. However, this technique requires the use of a scanning densitometer and is time consuming. An integral measurement requiring minimal analysis is desired.

## II. CONCEPT

An electron beam's PDD curve is characterized by a region of more or less uniform dose followed by a rapid falloff, especially in the range of 4 to 15 MeV.^4^ At depths greater than the electron range, only small doses are delivered. An ionization chamber under buildup provides a response that depends on PDD at the buildup depth. To first approximation, the electron PDD curve can be modeled as a pulse function. If a chamber is at a depth within the electron range, then it will respond with a “high” signal. If it is below the electron range, it will respond with “low” signal.

If a linear, cylindrical chamber is irradiated under a slab absorber of thickness greater than the electron range, it registers only a small signal. If the absorber is longitudinally shifted so that part of the chamber is exposed, then the chamber generates a partial signal. Since the fractional volume irradiated is equal to the fractional length exposed, the chamber signal will be roughly proportional to the distance along the chamber by which the absorber is shifted.

**Figure 1:**
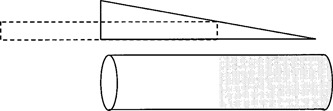
Measurement geometry showing high and low signal regions.

If the chamber is covered by a wedge‐shaped absorber, then each location along the chamber is shielded to a different radiological depth. However, the situation is analogous to that of the shifted slab as shown in Fig. 1. One segment of the chamber is shielded to a depth less than the electron range and the remaining segment is shielded to a depth greater than the range. The value of the range dictates what fraction of the chamber is irradiated. Thus chamber signal, relative to the unshielded signal, indicates the electron range and thus the electron energy. A wedge‐shaped absorber over a linear ionization chamber acts as a penetrameter, indicating increasing beam energy by an increase in net transmission. Net transmission is here considered to be ionization measured with the wedge in place to that measured with the wedge removed.

The absolute transmission of the wedge is offset by the absolute position of the wedge along the chamber. Shifts of the wedge along the chamber add or subtract the chamber signal as shifts of a slab would add or subtract the signal. The sensitivity of the wedge in discriminating energy is increased as the slope of the wedge is increased.

For validation, a cylindrical CT ionization chamber (RADCAL Model 10×5−3 CT/BNC,10.0−cm sensitive length) was selected as the detector. All measurements were made in air with the supplied acrylic buildup cap (${\frac{1}{2}}^{\prime \;\prime }$ outer diameter) and without backscatter.

Cerrobend was selected as the absorber material because it is readily available. The wedge was cast in a mold constructed of three pieces of expanded polystyrene. The length of the base was 11 cm, long enough to cover the length of the chamber. The thickness of the heel was 1 cm, which yields a wedge angle of approximately 5% $\left[\tan^{-1}(\frac{1}{11})=5.2{}^{\circ}\right]$. Small uncertainties in the slope of the wedge are not critical when a single wedge is to be used in a constancy measurement. The wedge was wider than the diameter of the ionization chamber.

## III. VALIDATION

The objective of validation was to demonstrate, through measurement, that a wedge‐shaped absorber can be used to detect the mean incident energy of a clinical electron beam and can thus be used to verify the constancy of that energy over time.

During these measurements, the toe of the wedge was approximately aligned to one end of the chamber and the wedge was not moved between measurements. The absolute ionization measured under the wedge depends on the absolute position of the wedge with respect to the chamber. Since the wedge was not precisely placed with respect to the chamber, the absolute ionization measured at specific energies is not meaningful and is thus not tabulated. When used to verify energy constancy as part of a quality assurance program, a wedge must be locked with alignment pins into a fixed and reproducible position.

The result, which is of interest, is the change of the signal generated in the ionization chamber as beam energy is changed. Measurements were made using the electron energies available on a VARIAN Clinac 18. A 15×15 cm cone was used to create an electron field larger than the ionization chamber. The base of the wedge was oriented perpendicular to the central axis of the beam at an SSD of 100 cm. The ionization chamber was located immediately behind the absorbing wedge. Expanded polystyrene was used to support the wedge and chamber and radiation was delivered with the accelerator gantry in a lateral position. For each available energy, ionization was measured with and without the wedge in place.

**Figure 2:**
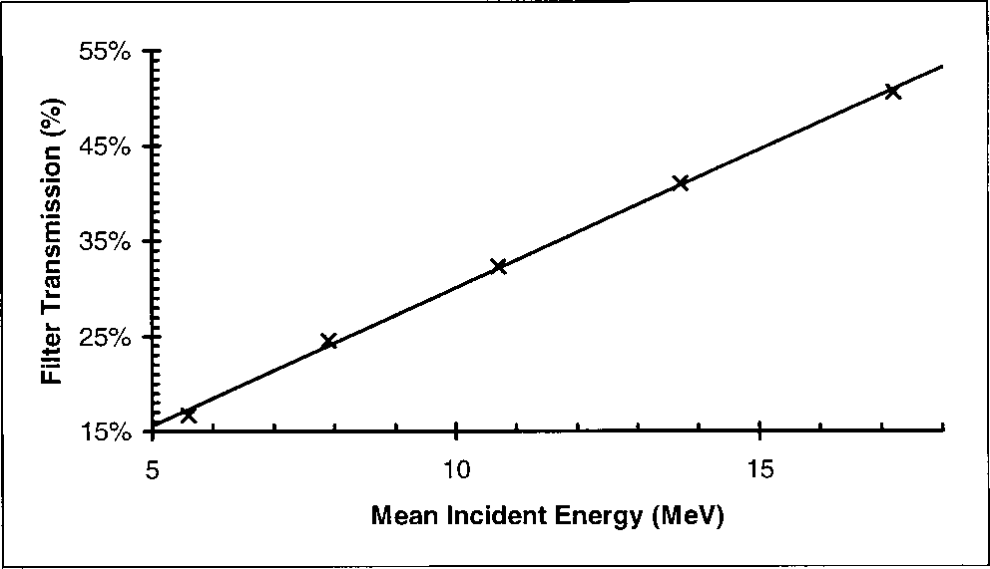
Energy dependence of net transmission.

These net transmission values resulting from these measurements are shown in Fig. 2. Transmission is seen to depend in a linear fashion on mean incident energy. The energy sensitivity, i.e., slope, of the response curve is 2.9%/MeV, which makes this method adequate for quality assurance purposes. Based on the definition of ${\bar{E}}_{0}$, a 2 mm shift in $d_{50}$ would result from an energy change of 0.466 MeV. Given the measured energy sensitivity, a 0.466‐MeV energy change would result (2.9%/MeV×0.466 MeV) in a 1.35% change in ionization ratio. This change would be readily discernible with a good quality electrometer.

## IV. CONCLUSION

Use of a wedge‐shaped absorber with a linear ionization chamber was shown to be a feasible alternative to the common method of verifying energy constancy of a clinical electron beam. This alternative method offers three advantages: (1) The measurement procedure need not be customized for each specific treatment unit; (2) only two measurement configurations (wedge in and wedge out) are required to check a cluster of energies; (3) the measurement result is a simple ionization ratio that can be directly interpreted in terms of the constancy of ${\bar{E}}_{0}$.
